# Fast Hologram Calculation Method Based on Wavefront Precise Diffraction

**DOI:** 10.3390/mi14091690

**Published:** 2023-08-29

**Authors:** Zimu Wang, Yilong Li, Zhenyan Tang, Zhaosong Li, Di Wang

**Affiliations:** 1 School of Instrumentation and Optoelectronic Engineering, Beihang University, Beijing 100191, China; 20373169@buaa.edu.cn (Z.W.); liyilong@buaa.edu.cn (Y.L.); 20373929@buaa.edu.cn (Z.T.); lzs1998@buaa.edu.cn (Z.L.); 2 State Key Laboratory of Virtual Reality Technology and Systems, Beihang University, Beijing 100191, China

**Keywords:** 3D display, holographic display, hologram

## Abstract

In this paper, a fast hologram calculation method based on wavefront precise diffraction is proposed. By analyzing the diffraction characteristics of the object point on the 3D object, the effective viewing area of the reproduced image is analyzed. Based on the effective viewing area, the effective hologram size of the object point is obtained, and then the accurate diffraction calculation from the object point to the wavefront recording plane (WRP) is performed. By calculating all the object points on the recorded object, the optimized WRP of the whole 3D object can be obtained. The final hologram is obtained by calculating the diffraction light field from the WRP to the holographic plane. Compared with the traditional method, the proposed method can improve the calculation speed by more than 55%, while the image quality of the holographic 3D display is not affected. The proposed calculation method provides an idea for fast calculation of holograms and is expected to contribute to the development of dynamic holographic displays.

## 1. Introduction

Based on the principles of light wave interference and diffraction, holographic 3D display technology can display images of real or virtual 3D objects suspended in space [1]. Since holographic 3D display technology can reproduce the complete wave-front information of 3D objects to the viewers, it is regarded as one of the most competitive naked-eye 3D display technologies [2]. In the calculation of holograms, 3D objects are usually represented in the form of points, polygons, or slices [3]. For different representations of 3D objects, there are different acceleration algorithms, such as point-based methods, polygon-based methods, and layer-based methods to calculate the hologram of 3D objects [4,5,6,7,8,9]. Moreover, the calculation of the hologram can be accelerated by using large-scale integrated circuits and deep learning [10,11,12]. However, holographic 3D display technology still has some technical difficulties and challenges, such as the narrow field of view [13], serious speckle noise [14], and slow calculation speed [15].

To address these problems, researchers have conducted a lot of innovative and optimized research in optical systems [16,17,18], materials [19,20], and algorithms [21,22]. However, the high-quality holographic 3D display based on the point source model still cannot be achieved with high efficiency. One of the most critical limitations is that the generation speed of the hologram cannot be significantly improved. Generating holograms of 3D objects is a time-consuming and complex process because the amount of data regarding the recorded 3D object is usually large, and the calculation process involves a lot of operations [23]. It should be noted that the hologram acceleration algorithm based on the point-based methods has been greatly developed in recent years [24]. The classical point-based methods are the look-up table method and the wavefront recording plane (WRP) method [25,26]. The look-up table method can quickly generate the hologram by superimposing the pre-calculated fringe patterns for each object point. However, the look-up table (LUT) method consumes a large amount of computer memory during hologram calculation. The novel look-up table (NLUT) only calculates and stores the primary fringe pattern of the object point, thus greatly reducing the amount of hologram calculation [27].

In order to reduce the storage space requirements of the LUT algorithm, researchers have introduced the separated look-up table (S-LUT) algorithm. This approach utilizes modulation factors in the X and Y directions as substitutes for the data stored in the hologram table, effectively reducing both the storage demands of the LUT and the number of data retrieval cycles. As a result, this method significantly enhances the calculation speed of hologram generation. To further diminish storage space utilization within the LUT and enhance hologram calculation efficiency, researchers have proposed the compressed look-up table (C-LUT) method. This algorithm comprises two key steps: firstly, the precomputation of X-Y direction modulation factors offline, which are subsequently stored in the table; secondly, during online operations, the extraction of X-Y direction modulation factors corresponding to the points representing the target object is performed from the table. These factors are then multiplied by the corresponding Z-direction modulation factors to generate the hologram. Although the C-LUT method does not entail a slow hologram calculation speed, it does introduce phase modulation errors approximated within the algorithm, necessitating subsequent phase correction. This introduces an added layer of complexity to the algorithm [28,29].

The WRP method records the complex amplitude information in the virtual plane close to the 3D object, which is subsequently diffracted and propagated to the hologram diffraction plane to obtain the hologram of the recorded object [30,31]. During the calculation of the hologram, the procedure commences by employing ray tracing techniques to calculate the complex amplitude distribution of each object point on the WRP. Subsequently, this distribution is propagated to the hologram plane through either Fresnel or angular spectrum diffraction methods. Given the proximity of the WRP to the 3D object, subject to diffraction limitations, the propagated object light wave is confined to a small region on the WRP. Consequently, the contribution area of the point source’s object light wave to the WRP is substantially limited, thereby resulting in a substantial reduction in the calculation complexity of the hologram generation. The initial step of the WRP method involves calculating the contribution of each point source to the WRP using ray tracing techniques, a process demanding extensive computational resources. Some researchers put forward an improved WRP method to reduce the hologram calculation time further by using the graphics processing units [32]. However, the reconstructed image has an effective diffraction area, and the hologram outside the effective diffraction area is a redundant calculation [33]. In the past, point-based methods rarely considered the effective diffraction area when calculating holograms, which resulted in the waste of extra calculation time.

With the rapid advancement of deep learning algorithms and iterative technology, tomographic methods rooted in deep learning or alternative iterative approaches have exhibited the ability to produce high-quality holograms quickly. However, it is important to note that this does not mean that the optimization algorithms based on point source or polygon methods are meaningless. Point source methods have unique advantages, such as their operational flexibility, their ability to represent the object surface characteristics, and their great potential in dealing with occlusion relationships and point cloud models.

In this paper, a fast hologram calculation method based on the wavefront precise diffraction is proposed. Different from traditional methods, we introduce a WRP between the object and the holographic plane to improve the calculation speed of the traditional NLUT method. The WRP records the complex amplitude distribution in the virtual plane close to the 3D object to cut down the diffraction distance from the 3D object point to the hologram plane. The 3D object consists of many object points, and the diffraction area of each object point is divided into the effective diffraction area and the ineffective area. The effective diffraction area contains all the information needed to reconstruct a complete image, while the ineffective diffraction area does not. The WRP is generated by analyzing the effective diffraction area of each object point and calculating the complex amplitude information corresponding to the effective diffraction area on the WRP. In this way, the accurate diffraction calculation from the object point to the WRP is realized. By superimposing the WRP information corresponding to the object point, the total complex amplitude information of the WRP is obtained. The final phase-only hologram is obtained by analyzing the diffraction of the total WRP to the hologram plane. In the proposed method, the calculated WRP of each object point is smaller than that of the traditional method because the complex amplitude information of the invalid diffraction region on the WRP is not calculated. Therefore, the proposed method has obvious advantages in calculation speed. The proposed method has a good display effect and broad application prospects.

## 2. Principle of the Method

The holographic generation algorithm based on point source models involves accounting for the impact of each individual point light source on the desired optical field when calculating holograms. Specifically, in the context of the NLUT algorithm, the final hologram is composed of the accumulation of sub-holograms corresponding to each point light source. The majority of calculation time within this algorithm is dedicated to addition-based calculations. As the pixel count of the 3D object increases, the frequency of addition-based calculations grows rapidly. Through the implementation of the WRP, we significantly reduce the resolution of the sub-holograms. This reduction substantially diminishes the frequency of addition-based computations, consequently leading to a remarkable enhancement in hologram generation speed.

**Figure 1:**
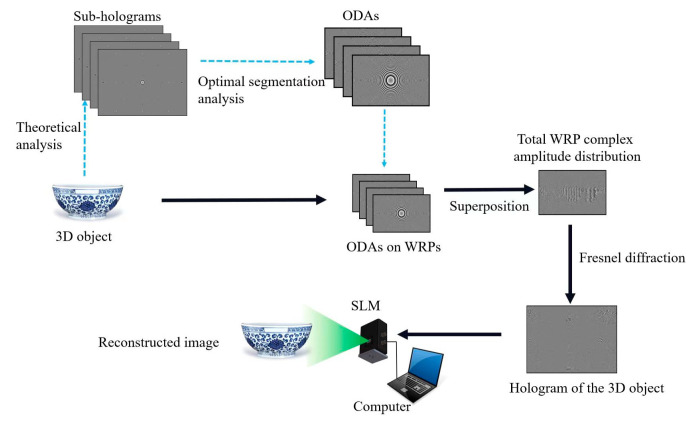
Process of the proposed method.

The proposed method consists of four steps, as shown in Figure 1. In the first step, the sub-holograms of each point of the 3D object are generated separately according to the Fresnel diffraction theory. In the second step, the optimal segmentation of each image object is performed to obtain the optimized diffraction area (ODA) of each sub-hologram. In the third step, the WRP is introduced and the accurate diffraction from the object point to the WRP is calculated. The complex amplitude distribution of the ODA on the corresponding WRP is superimposed to obtain the total complex amplitude information on the WRP. In the fourth step, the total complex amplitude distribution on the WRP is diffracted to the holographic plane to obtain the final hologram. After that, the final phase-only hologram is loaded on the phase-only spatial light modulator (SLM) to obtain the reconstructed image of the recorded 3D object. Since the final hologram is only calculated from the complex amplitude distribution of the ODAs on the WRP, the speed of hologram generation is greatly improved. Notably, the segment indicated by the blue dashed arrow represents a precomputed component, housing universally applicable sub-holograms that can be employed across hologram calculations for various 3D objects. During the generation of holograms for specific 3D objects, it suffices to extract the precomputed sub-holograms, directly calculating the optimized segmented sub-holograms on the WRP.

In the first step, the complex amplitude information of each object point on the holographic plane is calculated by the Fresnel diffraction formula, which can be expressed as follows:

(1) $$U(x,y)=\frac{\exp(2j\frac{\pi }{\lambda }z)}{j\lambda z}\int \int_{-\infty }^{+\infty }U_{0}(x_{0},y_{0})\exp\{j\frac{\pi }{\lambda z}[{\left(x-x_{0}\right)}^{2}+{\left(y-y_{0}\right)}^{2}]\}dx_{0}dy_{0}$$

where *U*(*x*, *y*) represents the complex amplitude information of the object point on the holographic plane, *λ* represents the wavelength, *z* represents the diffraction distance, and *U*_0_(*x*_0_, *y*_0_) is the complex amplitude distribution of the object point (*x*_0_, *y*_0_).

**Figure 2:**
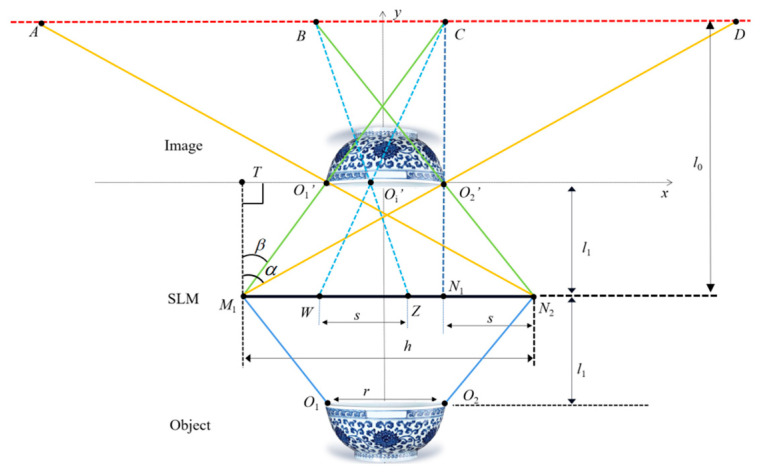
Analysis of the effective diffraction area.

In the second step, in order to conduct the accurate diffraction calculation, the optimal segmentation is first achieved by analyzing the effective diffraction regions of each point, resulting in the acquisition of the ODA. As shown in Figure 2, the depth slice plane *O*_1_*O*_2_ of the 3D object is taken as an example, and the leftmost and rightmost points on the depth slice plane are denoted as *O*_1_ and *O*_2_, respectively. The reconstructed image points of *O*_1_ and *O*_2_ are recorded as *O*_1_′ and *O*_2_′, respectively. The coordinate system is established with the line connecting *O*_1_′ and *O*_2_′ being the *x*-axis. The size of the SLM is *h*. The size of *O*_1_*O*_2_ is *r*, and the distance between the reconstructed image and the SLM is *l*_1_. The observation distance is *l*_0_. The diffraction region of the image point *O*_1_′ is *AC*, and the diffraction area of the image point *O*_2_′ is *BD*. Only in the *BC* where *AC* and *BD* are overlapped, the information of the reconstructed image is complete. Therefore, the *BC* is called the effective diffraction area. While in the other areas, such as *AB* and *CD*, the information of the reproduced image is missing, and the complete image cannot be reconstructed.

Based on the effective diffraction region *BC*, the maximum diffraction angle of the reconstructed image can be obtained as $\beta =\arctan(h-r)/2l_{1}$. By optimizing the sub-hologram of the object point of the 3D object, the ODAs on the sub-holograms that contribute to the reconstruction of the complete image are obtained. For example, the ODA of the rightmost point *O*_2_ is *N*_1_*N*_2_ and the size is *s*, which is smaller than the size *h* of its original sub-hologram *M*_1_*N*_2_. The size of the diffraction region *AC* is $l_{AC}=(l_{0}-l_{1})h/l_{1}$ and the size of the diffraction region *AB* is $l_{AB}=l_{0}r/l_{1}$, so the size of the effective diffraction region *BC* is $l_{BC}=l_{0}(h-r)/l_{1}-h$. According to the geometric relationship, the size of the ODA corresponding to the object point on the depth slice plane is $s=h-l_{0}r/(l_{0}-l_{1})$. Based on the above analytical calculations, it can be concluded that the ODA of the image point is smaller than *h*.

**Figure 3:**
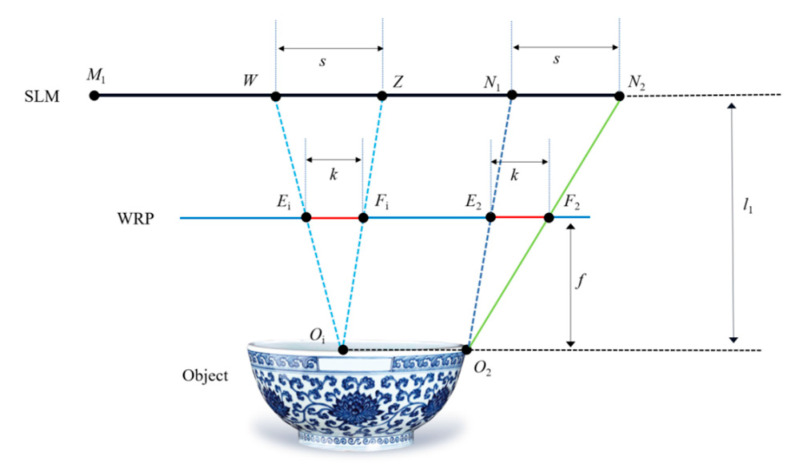
Accurate diffraction analysis of the WRP.

In the third step, the WRP is added between the holographic plane and the 3D object to further improve the calculation speed. The accurate diffraction area of the WRP is obtained by analyzing the effective diffraction area. The same depth slice plane of the 3D object corresponds to the same WRP, and different depth slice planes correspond to the different WRPs. As shown in Figure 3, based on the analysis of the effective diffraction region in the second step, the effective area on the WRP plane can be calculated for each object point on the depth slice plane *O*_i_*O*_2_. From the above analysis, it is clear that the size of the sub-hologram of each object point on the depth slice plane *O*_i_*O*_2_ is *s*. Therefore, when the distance from the depth slice plane to the WRP is *f*, according to the geometric relationship, the effective size *k* of the WRP corresponding to the object point can be obtained as follows:

(2) $$k=\frac{f}{l_{1}}\cdot (h-\frac{l_{0}}{l_{0}-l_{1}}r)$$

**Figure 4:**
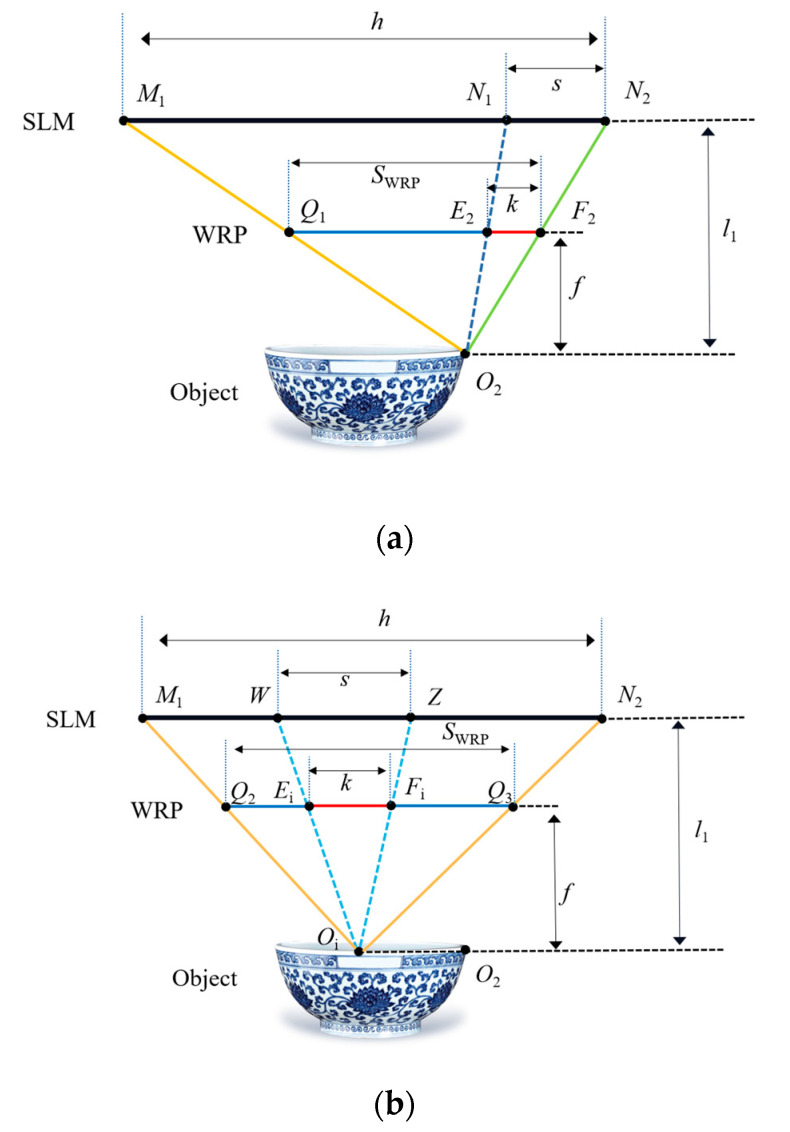
Comparison of the effective diffraction area of the WRP for the proposed method and the traditional method. (**a**) Effective area size of *O*_2_; (**b**) effective area size of the any point *O*_i_.

Then, the effective area of the WRP corresponding to the object point is superimposed, and the total WRP complex amplitude distribution can be obtained. When the traditional method and the proposed method are used, respectively, the comparison of the effective area of point *O*_2_ and any point *O*_i_ on the WRP in the same depth plane are shown in Figure 4. The sizes of the WRP effective areas *Q*_1_*F*_2_ and *Q*_2_*Q*_3_ for *O*_2_ and *O*_i_ object points calculated by the traditional method are as follows:

(3) $$S_{\mathrm{WRP}}=\frac{f}{l_{1}}\cdot h.$$

Based on Equations (2) and (3), it can be seen that *S*_WRP_ is greater than *k*. Compared with the traditional WRP calculation method, the proposed method greatly reduces the calculation volume and thus improves the generation speed of the hologram. It should be noted that the position of the WRP corresponding to each depth slice plane of the 3D object should be neither too far nor too close to the object point, otherwise the generation speed of the hologram and the quality of the reproduced image may be affected.

In the fourth step, the total WRP complex amplitude information is diffracted to the hologram plane to obtain the final hologram. Finally, the final phase-only hologram is loaded on the phase-only SLM, and the complete reproduced image can be seen when the hologram is illuminated by the reconstructed light.

## 3. Experiments and Discussion

**Figure 5:**
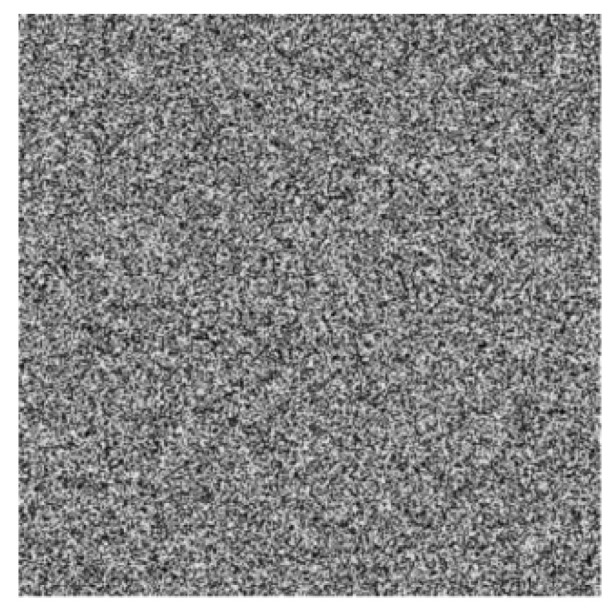
Random speckle pattern.

In the experiment, the laser wavelength was 532 nm. The resolution of the SLM was 1920 × 1080 and its pixel size was 6.4 µm. The viewing distance was set to 60 cm, and the distance between the WRP and the object was 12 cm. The diffraction distance was 30 cm. We used MATLAB 2022a software (9.12.0.1884302(R2022a)) for simulation. The memory of the computer was 16 GB and the processor was an Intel (R) Core (TM) i7-10750h (Thunderobot, Qingdao, China) (2.6 GHz). The proposed calculation method was also compared with the traditional NLUT method to prove its advantage in hologram calculation speed. Firstly, a random speckle pattern was selected as the recorded object for speed comparison, as shown in Figure 5.

### 3.1. Calculation of the Hologram

In the NLUT method, the final hologram was calculated by superimposing the sub-holograms of the object points directly. In the proposed method, by analyzing the effective diffraction area, the ODA of each sub-hologram was obtained, and then the effective area on the WRP could be calculated. By superimposing the effective area of each object point on the WRP, the total WRP could be obtained, and finally the hologram of the recorded object was generated. The calculation speed is shown in Table 1. From the results we can see that the proposed method can effectively improve the calculation speed and save calculation time. When the resolution of the random speckle patterns is 300 × 300, 350 × 350, 400 × 400, and 450 × 450 and 500 × 500, the calculation speed improvement ratios by using the proposed method are 53.48%, 55.37%, 53.21%, 48.6%, and 48.8%, respectively.

**Figure 6:**
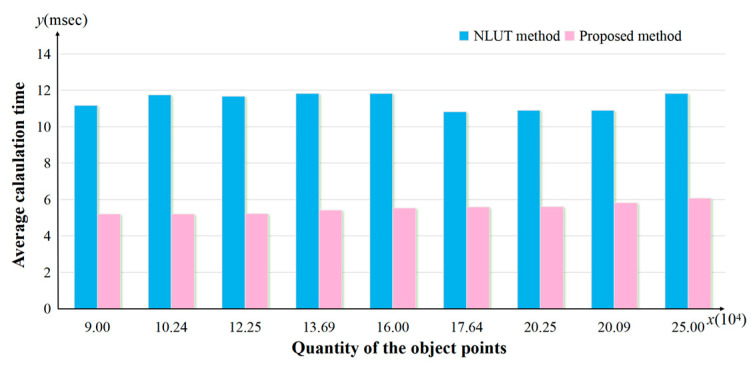
Average calculation time for an object point.

Figure 6 shows the average generation time variations using the proposed method and the NLUT method when the object points quantity changes. When the hologram is calculated by MATLAB or another software, the addition calculation takes up most of the calculation time, and the amount of addition calculation depends on the resolution of the sub-hologram. Thus, the resolution of the sub-hologram plays a key role in the average calculation time of a point. Because the resolution of the proposed method is much smaller than that of the NLUT method, the calculation speed of the proposed method is increased by about 50%.

### 3.2. Holographic Reconstruction

**Figure 7:**
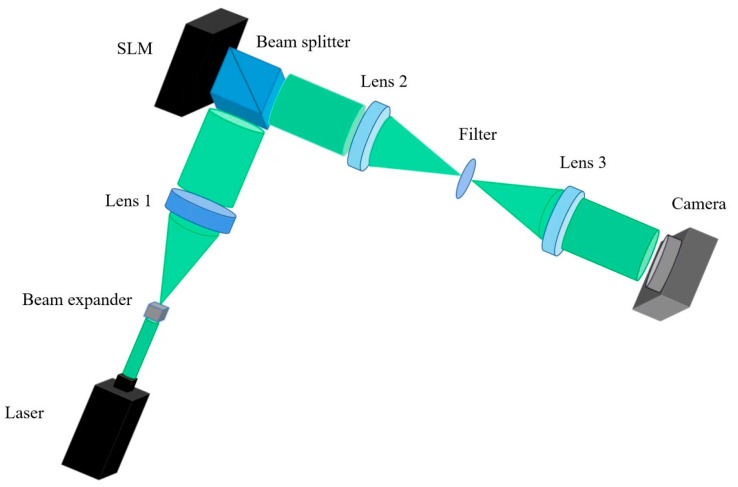
Optical path diagram for experimental verification.

In order to show that the proposed calculation method can reduce the calculation time without reducing the quality of the reconstructed image, we set up an optical path diagram for experimental verification, as shown in Figure 7. Two 2D objects and a 3D object were selected for the experiment, respectively. The green laser irradiates the SLM after passing through the beam expander, lens 1, and the beam splitter (BS). The BS had a size of 25.4 mm × 25.4 mm × 25.4 mm. A 4*f* system consisting of the lens 2, filter, and lens 3 was used to filter out the stray light. All three lenses had a focal length of 30 cm. The filter was produced by Daheng Company (GCM-5701M, Beijing, China). When considering the integration of the 4*f* system, its profound impact on image quality becomes evident. Particularly, the shift towards a lensless configuration introduces a scenario where the holographic diffraction field is susceptible to the intrusion of both zero-order and high-order diffraction light interferences. This presence of interference components can significantly compromise the fidelity of holographic reconstruction. Notably, the adoption of the 4*f* system holds the ability to effectively mitigate these interferences effectively through the utilization of spectral plane filtering mechanisms. This intervention ultimately contributes to the preservation and enhancement of the overall quality of the holographic reconstruction. Finally, the reproduced image can be captured by the camera.

**Figure 8:**
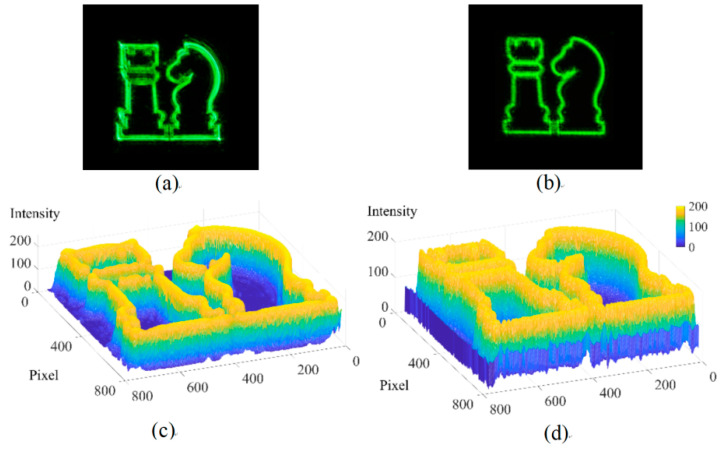
Reproduced results of the 2D object. (**a**) Reproduced image of the NLUT method; (**b**) reproduced image of the proposed method; (**c**) light intensity distribution of the NLUT method; (**d**) light intensity distribution of the proposed method.

Firstly, a 2D object ‘Chess’ with a resolution of 800 × 800 was utilized as the recorded object. The resolution of the hologram was 1920 × 1080. The proposed method exhibited a generation time of 943.485 s, while the NLUT method takes 1911.490 s. The proposed method boasts a calculation speed improvement ratio of 50.65%. The reconstructed results and light intensity distribution of the NLUT method and the proposed method are shown in Figure 8. The proposed method can reproduce all the details of the recorded object, which indicates that the optimized segmentation and the acceleration mode of introducing the WRP adopted in the proposed method have not lost any information about the object. The PSNR of the NLUT method and the proposed method are 10.42 dB and 10.62 dB, respectively.

**Figure 9:**
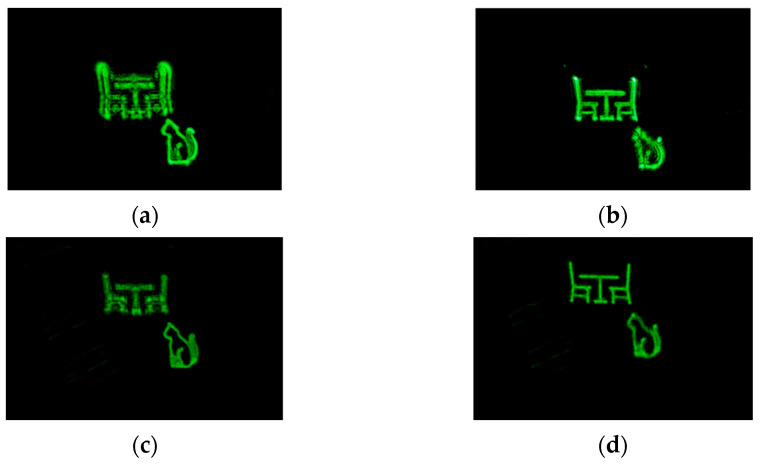
Reproduced results of the 3D object. (**a**) Reproduced image of the NLUT method when the ‘cat’ is focused; (**b**) reproduced image of the NLUT method when the ‘chairs’ is focused; (**c**) reproduced image of the proposed method when the ‘cat’ is focused; (**d**) reproduced image of the proposed method when the ‘chairs’ is focused.

In addition, we selected ‘chairs’ and ‘cat’ located at different depths as the 3D objects for experiments, which proved that the proposed method could realize holographic reconstruction of the 3D object. The ‘chairs’ and ‘cat’ were 0.12 m and 0.2 m away from the hologram plane, respectively, and their resolution was 750 × 750. The ‘chairs’ and the ‘cat’ were separately focused on two different depth planes, as shown in Figure 9. Figure 9a is the reproduced image of the NLUT method when the ‘cat’ is focused and Figure 9b is the reproduced image of the NLUT method when the ‘chairs’ is focused. Figure 9c,d shows the reproduced image of the proposed method when the ‘cat’ and the ‘chairs’ are focused, respectively. It can be seen that the reproduced images generated by the proposed method can focus correctly at different depths.

Compared with the NLUT method, the proposed method has obvious advantages in the calculation speed of the hologram and can ensure that the imaging quality of the reconstructed image is not affected. For the 3D objects, the proposed method has more obvious depth effects than the NLUT method. As can be seen from Figure 9, when ‘cat’ is in focus, the object in another depth plane is obviously out of focus. The defocusing effect of the proposed method in Figure 9 is stronger than the NULT algorithm, that is to say, its depth sensitivity may be higher.

Our proposed method operates by eliminating redundant regions within the sub-holograms, thereby increasing the calculation speed of the hologram generation. It is worth noting that these redundant regions do not contribute meaningfully to the reconstruction of the 3D object. However, despite their lack of substantive impact on object reconstruction, these eliminated regions can still exert an influence on the overall intensity distribution of the optical field. Consequently, with the same experimental system, our method exhibits a slightly reduced overall luminance in comparison to traditional methodologies. In the future, we will continue to study the depth information of the proposed method to obtain better 3D display results.

## 4. Conclusions

In this paper, a fast hologram calculation method based on the wavefront precise diffraction is proposed. By analyzing the effective viewing region of the reconstructed image, the effective hologram size of each object point is obtained, and then the accurate diffraction calculation from the object point to the WRP is realized. Compared with the traditional WRP methods, the size of the WRP in the proposed method is smaller. The final hologram is obtained by analyzing the diffraction from the WRP to the holographic plane. The proposed method realizes the accurate calculation of the WRP and effectively improves the calculation speed of the hologram. The novel contribution of our method revolves around substantially mitigating one of its primary limitations, namely calculation speed. Through this innovation, we aspire to render the point-source holographic algorithm more competitive and versatile, thereby broadening its applicability and relevance within the realm of holographic display technology.

## Figures and Tables

**Table 1:** Hologram calculation time of the proposed method and the NLUT method.

Method	Resolution of the Object	Calculation Time of the Hologram (s)	Average Calculation Time for an Object Point (ms)
NLUT method	300 × 300	1006.070	11.179
	350 × 350	1431.290	11.684
	400 × 400	1892.773	11.830
	450 × 450	2209.453	10.911
	500 × 500	2958.002	11.832
Proposed method	300 × 300	468.058	5.200
	350 × 350	638.831	5.215
	400 × 400	885.600	5.535
	450 × 450	1135.049	5.605
	500 × 500	1515.477	6.062
